# Transition of care in post-hospitalization patients due to covid-19 in a hospital in northeastern Brazil

**DOI:** 10.1590/0034-7167-2023-0030

**Published:** 2023-10-09

**Authors:** Marianny Nayara Paiva Dantas, Evelyn Silva de Sousa, Sarah Lyandra Furtado Faustino, Isabelle Campos de Azevedo, Viviane Euzébia Pereira Santos

**Affiliations:** I Universidade Federal do Rio Grande do Norte. Natal, Rio Grande do Norte, Brazil

**Keywords:** Patient Discharge, Self-Management, Transitional Care, Hospitalization, Coronavirus Infections, Alta del Paciente, Automanejo, Cuidado de Transición, Hospitalización, Infecciones por Coronavirus, Alta do Paciente, Autogestão, Cuidado Transicional, Hospitalização, Infecções por Coronavírus.

## Abstract

**Objective:**

To analyze the transition of care for post-hospitalization patients due to covid-19 in a hospital in northeastern Brazil.

**Methods:**

Quantitative, cross-sectional, descriptive, and analytical study carried out between 2020 and 2021. The sample had 78 patients. Data collection took place by telephone with the support of a sociodemographic questionnaire and the care transition assessment instrument (Care Transitions Measure).

**Results:**

The average length of stay was 24.04 days. The average score for care transition was 71.68 (±11.71). “Self-management training” and “Understanding of medications” had higher averages, 75.15 (±13.76) and 74.10 (±16.20).

**Conclusions:**

The average length of stay was 24.04 days. The average score for care transition was 71.68 (±11.71). “Self-management training” and “Understanding of medications” had higher averages, 75.15 (±13.76) and 74.10 (±16.20).

## RESUMO

**Objetivo:**

Analisar a transição do cuidado de pacientes pós-internação por covid-19 realizada em um hospital no Nordeste brasileiro.

**Métodos:**

Estudo quantitativo, transversal, descritivo e analítico realizado entre 2020 e 2021. A amostra contou com 78 pacientes. A coleta de dados ocorreu por telefone com apoio de um questionário sociodemográfico e do instrumento de avaliação da transição de cuidados (*Care Transitions Measure*).

**Resultados:**

O tempo médio de internação foi de 24,04 dias. A pontuação média para a transição do cuidado foi 71,68 (±11,71). O “Treinamento de autogestão” e o “Entendimento sobre medicações” obtiveram maiores médias, 75,15 (±13,76) e 74,10 (±16,20).

**Conclusões:**

A qualidade da transição do cuidado de pacientes da pós-internação por covid-19 foi satisfatória na instituição participante. Entretanto, a valorização das preferências do paciente e família para o autogerenciamento da doença bem como o plano de cuidados pós-alta precisam ser aprimorados.

## RESUMEN

**Objetivo:**

Analizar la transición del cuidado de pacientes postinternación por Covid-19 realizada en un hospital en el Nordeste brasileño.

**Métodos:**

Estudio cuantitativo, transversal, descriptivo y analítico realizado entre 2020 y 2021. La muestra se compuso de 78 pacientes. La recolecta de datos hecha por teléfono con apoyo de una encuesta sociodemográfica y del instrumento de evaluación de la transición de cuidados (Care Transitions Measure).

**Resultados:**

El tiempo mediando de internación fue de 24,04 días. La puntuación mediana para la transición del cuidado fue 71,68 (±11,71). El “Entrenamiento de automanejo” y el “Entendimiento sobre medicaciones” obtuvieron mayores medianas, 75,15 (±13,76) y 74,10 (±16,20).

**Conclusiones:**

La calidad de la transición del cuidado de pacientes de la postinternación por Covid-19 fue satisfactoria en la institución participante. Entretanto, la valorización de las preferencias del paciente y familia para el automanejo de la enfermedad así como el plan de cuidados postalta precisan ser perfeccionados.

## INTRODUCTION

Severe Acute Respiratory Syndrome Coronavirus 2 (SARS-CoV-2) was first identified in China in 2019 and has been classified as the causative agent of Coronavirus Disease 2019 (covid-19). Due to the high transmission potential and increase in the number of cases worldwide, the disease was considered a Public Health Emergency of International Concern (PHEIC); and, in March 2020, it was declared a pandemic state by the World Health Organization (WHO)^(1-2)^.

Most of covid-19 is manifested by mild flu-like symptoms, however the WHO^(3)^ points out that about 15% of patients evolve to hospitalization due to the worsening of their conditions and the need for oxygen therapy; of these, 5% require support in the Intensive Care Unit (ICU).

In its severe form, covid-19 can lead to functional disorders, such as fatigue, muscle weakness, dyspnea, neurological deficits, among others, which tend to last after hospitalization and result in home care and dependency^(4-5)^.

In this sense, preparing the patient for assistance at home or in other areas of the health network allows overcoming the fragmentation of care and contributes to the integrality of the service provided. Thus, it is necessary to develop strategies that seek improvements in the transition of care^(6)^.

The transition of care refers to a set of actions that aim to guarantee assistance between the different levels or places where health promotion takes place, including the home^(7)^. Thus, its correct implementation benefits both the patient and the health institution, due to the significant reduction in the number of readmissions, in addition to improving the quality of life of individuals and minimizing hospital costs^(8)^.

However, for its proper implementation, it is necessary to have multidisciplinary participation, in which post-hospitalization guidelines should be prioritized. It is at this moment that doubts, perceptions and anxieties are clarified, in order to promote the autonomy of the subjects and prevent injuries after discharge^(9)^.

In addition, another necessary aspect for the success of the transition of care is the involvement of the patient and his family, which must occur through awareness-raising and health education actions, developed based on the weaknesses and potential of the patient in his health-disease process^(10)^.

In view of this, investigating the post-discharge care transition strategies for patients with covid-19 can contribute with information for the qualification of professional practice and health services; moreover, such an investigation can help in understanding the theme, since, although recommended, the transition of care is little discussed in Brazil^(10)^.

## OBJECTIVE

To analyze the transition of care for post-hospitalization patients due to covid-19 in a hospital in Northeast Brazil.

## METHODS

This work was presented during the 73rd Brazilian Congress of Nursing (CBEn), promoted by the Brazilian Association of Nursing (ABEn). In addition, he received the “Honorable Mention - 2nd Place” of the Laís Neto dos Reis Award.

### Ethical aspects

The study is part of a multicenter project entitled “Assessment of Nursing Care for Patients with Covid-19 in Brazilian University Hospitals”, which involves ten federal educational institutions in Brazil. It is funded by the National Council for Scientific and Technological Development (CNPQ). The work was conducted in accordance with international and national ethics guidelines and was approved by the Ethics and Research Committee of the Federal University of Rio Grande do Norte (UFRN). It should be noted that the Declaration of Free and Informed Consent was obtained from all individuals involved in the study through verbal communication during phone calls.

### Type of study, place, and period

Descriptive, cross-sectional study with a quantitative approach, based on the recommendations of Strengthening the reporting of observational studies in epidemiology (STROBE)^(11)^. Its setting was the inpatient unit for patients with covid-19 at a teaching hospital located in Northeast Brazil, between April 2020 and December 2021.

### Population: inclusion and exclusion criteria

The study included 78 patients who were discharged from the inpatient unit for patients with covid-19 at the aforementioned institution. The number of participants was intentionally defined, in which a convenience sample of approximately 10% of the number of monthly discharges that occurred during the period from April 2020 to December 2021. The total number of beds in the institution was considered: 231 active beds and 17 beds for covid 19.

The inclusion criteria for patients were: being 18 years old or older; have fluency in Brazilian Portuguese and have had a minimum period of 72 hours of hospitalization, due to the greater chances of having undergone procedures during that time. Patients unable to participate were excluded because they were weak or distressed, among other reasons, such as pain or in the immediate postoperative period.

### Study protocol

Data collection was performed using the Care Transitions Measure^®^ (CTM) instrument, which was developed in 2002 in the United States of America (USA). It aims to assess the quality-of-care transition between different health care services from the patient’s perspective. It has two versions, the CTM-15, with 15 statements; and CTM-3, with only three statements^(12)^.

In its two versions, it was translated and validated for the Brazilian reality in 2016, ensuring its reliability and content. For the study, the CTM-15 was chosen, since it has 15 items separated by four factors, namely: Factor 1 - Self-management training (items 4, 5, 6, 8, 9, 10 and 11); Factor 2 - Understanding about medications (items 13, 14 and 15); Factor 3 - Assured preferences (items 1, 2 and 3); and Factor 4 - Care plan (items 7 and 12)^(12-13)^.

The CTM-15 was used by telephone, within a period of up to 30 days after hospital discharge. The calls were made by the same collector, who was previously trained. It is noteworthy that, at the time of telephone contact, the Declaration of Free and Informed Consent was obtained verbally by the patients; in addition, the questions were asked slowly, clearly, and objectively.

The instrument’s questions are Likert-type, with five options: Strongly Disagree (1 point); Disagree (2 points); Agree (3 points); Strongly agree (4 points). The fifth option, however, ‘I don’t know/I don’t remember/does not apply’’ (0 points), is not included in the score count.

The score is calculated by the sum of the values referring to the answers divided by the number of questions answered. After that, it must be transformed into a linear scale ranging from 0 to 100, in which higher values represent more structured care transition flows, using the formula: [(score-1)/3] * 100^(13)^.

### Data analysis and statistics

Data were analyzed using descriptive and inferential statistics using the Statistical Package for the Social Sciences (SPSS), version 25.0 for Windows. Categorical variables were represented by absolute and relative frequency; quantitative variables, mean and standard deviation, median and interquartile range (P50 [P25; P75]) and range (minimum and maximum).

For internal consistency, Shapiro-Wilk statistics were performed, in addition to the Shapiro-Wilk normality test to verify the distribution of quantitative variables (CTM Factor Scores). Thus, the comparison of the distributions of the scores of the CTM factors between the categories of sociodemographic variables was performed using non-parametric tests (Mann-Whitney or Kruskal-Wallis test).

In addition, Spearman’s correlation was performed to verify the degree of relationship between hospitalization variables and CTM factors. When significant, the intensity of the correlation can be classified as: weak, from 0 to 0.3; regular, from 0.4 to 0.6; strong, from 0.6 to 0.9; and very strong, from 0.9 to 1.0. The significance level adopted was 0.05^(14)^.

## RESULTS

Of the 78 study participants, 41 (52.0%) are male, 39 (50.0%) have incomplete primary and secondary education, and 34 (46.6%) have a family income of up to R$ 2,090. These and the other categorical variables are shown in Table 1. The mean total length of stay in the hospital was 24.04 days (23.12); and, in the Intensive Care Unit (ICU), it was 8.30 days (14.45).

**Table 1:** Characterization of the sample of patients affected by covid-19, Natal, Rio Grande do Norte, Brazil, 2022

Categorical variables	n (%)
Sex	
Male	41 (52.0)
Female	36 (46.8)
Other	1 (1.2)
Educational level	
No education and less than a year of study	5 (6.4)
Primary Education + incomplete High School	39 (50.0)
High School Complete + University Education	34 (43.6)
Race	
Brown	45 (57.7)
White	21 (26.9)
Black	10 (12.8)
Others	2 (2.6)
Family income	
Up to BRL 2,090	34 (46.6)
BRL 2,090 to BRL 5,225	31 (42.5)
More than BRL 5,226	8 (8.2)
No income	5 (2.7)
Use of invasive mechanical ventilation	
No	62 (79.5)
Yes	16 (20.5)
Smoking	
Non-smoking	58 (74.4)
Ex smoker	19 (24.4)
Smoker	1 (1.2)

Table 2 presents the comorbidities and symptoms reported by the participants, of which 40 (51.3%) had cardiovascular disease (CVD); and 39 (50.0%), systemic arterial hypertension (SAH). Furthermore, regarding the most frequent symptoms, 60 patients had cough (76.9%); 53 (67.9%) shortness of breath; 52 (66.7%), fever; and 51 (65.4%), fatigue.

**Table 2:** Description of comorbidities and symptoms of patients affected by covid-19, Natal, Rio Grande do Norte, Brazil, 2022

Comorbidity	n (%)
Chronic respiratory disease	
No	63 (80.8)
Yes	15 (19.2)
Systemic arterial hypertension	
No	39 (50.0)
Yes	39 (50.0)
Cardiovascular diseases	
No	38 (48.7)
Yes	40 (51.3)
Diabetes mellitus	
No	56 (71.8)
Yes	22 (28.2)
Kidney diseases	
No	57 (73.1)
Yes	21 (26.9)
Obesity	
No	63 (80.8)
Yes	15 (19.2)
Cancer	
No	72 (92.3)
Yes	6 (7.7)
Fever	
No	26 (33.3)
Yes	52 (66.7)
Fatigue	
No	27 (34.6)
Yes	51 (65.4)
Shortness of breath	
No	25 (32.1)
Yes	53 (67.9)
Cough	
No	18 (23.1)
Yes	60 (76.9)
Loss of smell and taste	
No	41 (52.3)
Yes	37 (47.7)
Headache	
No	41 (52.3)
Yes	37 (47.7)
Chronic respiratory disease	
No	63 (80.8)
Yes	15 (19.2)
Body pain (muscles and joints)	
No	32 (41.0)
Yes	46 (59.0)
Nausea and vomiting	
No	48 (61.5)
Yes	30 (38.5)
Diarrhea	
No	43 (55.1)
Yes	35 (44.9)

Table 3 shows the result of the CTM-15. It is observed that the total score obtained an average of 71.68 (±11.71), in which Factor 1 (Self-management training) obtained the highest average, 75.15 (±13.76); and Factor 4 (Care Plan) the lowest, 65.60 (±24.52). Furthermore, regarding the internal consistency of the factors, Cronbach’s alpha coefficient for the instrument resulted in 0.869, in which Factor 1 (Self-management training) obtained 0.866, the highest value.

**Table 3:** Distribution of means and standard deviation of the total CTM-15 score and by factors, as well as Cronbach’s alpha, Natal, Rio Grande do Norte, Brazil, 2022

CTM-15 Score	Mean (SD)	Cronbach's alpha
**Total**	71.68 (±11.71)	0.869
**By factors**		
CTM - Factor Score 1	75.15 (±13.76)	0.866
CTM - Factor Score 2	74.10 (±16.20)	0.562
CTM - Factor Score 3	71.87 (±16.66)	0.781
CTM - Factor Score 4	65.60 (±24.52)	0.521

Table 4 shows the mean and standard deviation obtained for each CTM-15 item. The items with the highest scores were 14 and 5, which, respectively, belong to Factor 2 - Understanding about medications and Factor 1 - Self-management training’. On the other hand, the lowest averages were those of item 15 (Factor 2 - Understanding about medications) and item 4 (Factor 1 - Self-management training).

**Table 4:** Items by factors, mean and standard deviation of the CTM-15 items, Natal, Rio Grande do Norte, Brazil, 2022

Item	Factor	Mean (SD)
1. Agreed with the health team on goals for their health and how they would be achieved.	3	63.54 (±10.00)
2. Preferences considered to decide health needs.	3	64.36 (±13.15)
3. Preferences considered to decide where health needs are met.	3	61.54 (±12.80)
4. Had information they needed for self-care.	1	66.58 (±12.70)
5. Clearly understands how to take care of health.	1	67.34 (±11.18)
6. Understands warning signs and symptoms.	1	65.32 (±10.48)
7. Received a written plan of care.	4	61.04 (±15.86)
8. Understand what improves or worsens their health condition.	1	65.32 (±9.45)
9. Understands what their responsibility is.	1	66.58 (±9.99)
10. Feel confident that they know what to do.	1	62.28 (±12.81)
11. Feel confident that they can do what is required.	1	61.77 (±10.22)
12. Received a written list of appointments or exams.	4	57.47 (±19.31)
13. Understands the reason for taking the medication.	2	66.40 (±11.47)
14. Understands how to take medications.	2	69.46 (±10.58)
15. Understand the side effects of medications.	2	57.33 (±16.87)

As for the results of the association between age group, length of hospital stays and the CTM-15 factors, it can be seen that there was a weak association between the days of hospitalization (total and ICU) and the instrument factors (Table 5).

**Table 5:** Association between age group, length of stay and CTM-15 factors, Natal, Rio Grande do Norte, Brazil, 2022

	CTM			
	**Factor 1**	**Factor 2**	**Factor 3**	**Factor 4**
	**r (P)**	**r (P)**	**r (P)**	**r (P)**
Age	-0.052 (0.649)	-0.060 (0.614)	-0.077 (0.505)	0.144 (0.208)
Days of hospitalization (total)	-0.003 (0.980)	0.059 (0.615)	-0.042 (0.716)	0.175 (0.124)
Days of ICU stay	-0.039 (0.740)	0.077 (0.520)	-0.086 (0.462)	0.173 (0.134)

* r = Spearman Correlation Coefficient; + p-value = significance level.

## DISCUSSION

The results of the present study made it possible to analyze the transition from care at hospital discharge to the home of patients who had covid-19, with the help of the CTM-15, which, worldwide, is one of the most used instruments for this type of evaluation^(15)^. In addition, it should be noted that the transition of care is a unique and individual moment for each patient, therefore it must be carried out according to the specificities of each one.

The characterization of the sample of patients is in line with that found in a study^(16)^, in which a higher prevalence of males was observed in the post-discharge of patients affected by covid-19. However, in another research^(17)^, it was identified that the variable “gender” did not influence the probability of survival from this disease, which probably also does not interfere with the transition of care. In addition, there was a higher prevalence of brown people among users who were discharged and participated in the care transition process, although other studies^(18-19)^ point out that this population was the one that most evolved to death from the disease.

Regarding education, most patients have completed at least elementary school. It is emphasized that having some level of education has a direct impact on the fact that the patient recovers correctly from covid-19, as having the ability to understand information about the disease is reflected in greater self-care and improved prognosis^(19)^. Furthermore, it appears that, among the population studied, the patients had some morbidity, among which hypertension and other cardiovascular diseases stand out. This profile is consistent with that of the population that survived the disease, since multimorbidity increases the risk of worsening, hospitalization, and death from covid 19^(19)^. The presence of chronic non-communicable diseases associated with covid-19 is responsible for increasing the risk of death from this condition by 9.44%; and, in the Brazilian context, the highest mortality rates occurred among patients with CVD, which is in line with the profile of patients in this research^(17,19)^

It was verified that the average score of the CTM-15 was 71.68, which points to an average considered positive. Since the instrument does not have an established cutoff point, this finding is carried out in a comparative way with other studies, which ranged between scores of 82.4 and 76.4^(20-21)^ and adopted scores above 71.0 as satisfactory. Therefore, these values help guide preparations and improvements by the health team in relation to the transition of care, both in management and in care.

The CTM-15 Factor 1 score, “Self-management training”, obtained the best score among the instrument’s factors and high internal consistency between the item’s responses, which indicates quality in the care transition. Health education actions, promoting self-management, are described in the literature^(7,9)^ as those that are practiced by nurses and other health professionals more frequently in the transition from hospital to home. Therefore, the effectiveness of self-management training allows the patient and family to deal with a new health status, as well as reducing the possibility of readmissions^(22)^.

The CTM-15 Factor 2 score, “Understanding about medications”, also reached an average value indicative of quality in the transition of care. Medication conciliation at discharge is of great relevance for the continuity of treatment and successful recovery of the patient with covid 19, given that the systemic impairment caused by the disease may require the use of multiple drugs, such as antitussives, bronchodilators, antibiotics, and even high-alert medications, such as anticoagulants ^(22)^.

Factor 4, related to the “Care plan”, reached a score below the general average, which is in line with a similar Brazilian study^(23)^ and implies the relevance of actions that can improve the quality of this scope. It is necessary for the multidisciplinary team to be able to prepare a discharge plan including information about the disease, guidance on medications, care techniques and, above all, allow clarification of doubts.

This requires the individual’s proper self-management of their health, in addition to the exchange of knowledge between professionals, patients and family members since the task of caring can seem complex in the face of the absence of the necessary skills and a serious illness such as covid-19^(24)^. However, it is a new disease; and, at first, there was little scientific evidence about its form of action and treatment, which interfered in the care of patients during hospitalization as well as in the preparation for discharge.

It is important to emphasize the role of other Health Care Networks (HCN), especially Primary Health Care (PHC), as a constituent part of the effective transition of care. It is necessary for hospitals to articulate with these services so as not to have fragmentation of the assistance provided, since, in the community, there is the promotion of home visits and progressive follow-up of the patient, with a view to verifying their evolution and demands that arise^(25)^.

Factor 3, “Assured preferences”, scored as the second lowest score and converges with a study^(26)^ involving 50 patients from a US hospital. The role of patients and family members in the health-disease process is essential for proper post-discharge self-management. For this to happen, the multidisciplinary team must promote space for listening and debate, in order to welcome the opinions and positions that the patient-caregiver binomial needs. Thus, autonomy, an important ethical principle recognized in health, positively interferes with the continuity of care.

There was also a weak association between the days of hospitalization (total and ICU) and the MTC factors 15. Therefore, it can be deduced that the length of hospitalization did not negatively affect the quality of continuity of care. Research carried out in Spain^(11)^ inferred a correlation between “patient satisfaction with the transition of care” and “length of stay”. This fact can be explained by the greater technological apparatus that these environments have, in addition to the recommendations and daily care that allow greater chances of recovery and cure^(27)^. It is noteworthy that the results of this study did not associate between age group and the quality-of-care transition.

It was found that Cronbach’s alpha coefficient of the CTM-15 was considered satisfactory (0.869), which indicates good internal consistency of the instrument; this result is similar to studies in Brazil (0.850) and China (0.890)^(14,28)^.

Finally, it should be noted that the transition of care is still not formalized in the current reality. This can be evidenced by the lack of policies and programs that, throughout the territory, encourage its practice. Added to this fact, covid-19 is still considered a recent disease, and there is little information about its correlation with continuity of care. Thus, all these aspects end up making it difficult to fully understand the transition of care and can negatively impact the healing and recovery of affected patients.

### Study limitations

As a limitation of the study, we can mention the difficulty of telephone contact with some of the patients in the sample, since some felt insecure answering the requested questions and/or suspected that it might not be scientific research, but a scam.

### Contributions to the area of health

As contributions to the area, this study allows health professionals to expand their knowledge about the transition of care, in order to assist in the implementation of this process in health services. Transitional care allows the patient to be the center of care, while valuing the co-participation of the family and/or caregivers during hospitalization and post-discharge. Thus, surveys such as this one can collaborate so that information about the transition of care reaches society as a whole, not just health workers.

## CONCLUSIONS

By using the CTM-15, this study demonstrated that the transition of care for patients affected by covid-19 who are in post-hospitalization was satisfactory at the participating institution. Items related to guidelines on self-management of care and understanding of medications showed more favorable points to qualify the transition of care. However, there were still weaknesses that need to be improved, such as factors related to the preferences of patients and families for managing the disease and the care plan made, which must be agreed between professionals, patients, and family members while still in the hospital unit.

Therefore, further investigations are recommended in order to broadly understand and, in different scenarios, the transition of care for patients affected by covid-19 who are post-hospitalization. In addition, health professionals, managers, and the population in general need to debate and articulate so that, in fact, there are even more significant improvements in this environment.

## Data Availability

https://doi.org/10.48331/scielodata.20ZPIG
